# Clinical phenotype modulates brain’s myelin and iron content in temporal lobe epilepsy

**DOI:** 10.1007/s00429-021-02428-z

**Published:** 2021-11-24

**Authors:** Elisabeth Roggenhofer, Evdokia Toumpouli, Margitta Seeck, Roland Wiest, Antoine Lutti, Ferath Kherif, Jan Novy, Andrea O. Rossetti, Bogdan Draganski

**Affiliations:** 1 LREN, Centre for Research in Neuroscience, Department of Clinical Neurosciences, Lausanne University Hospital and University of Lausanne, Mont Paisible 16, 1011 Lausanne, Switzerland; 2 EEG and Epilepsy Unit, Department of Neurology, Department of Clinical Neurosciences, University Hospitals and Faculty of Medicine Geneva, Geneva, Switzerland; 3 Support Center for Advanced Neuroimaging, Institute for Diagnostic and Interventional Neuroradiology, University Hospital Inselspital, University of Bern, Bern, Switzerland; 4 Service of Neurology, Department of Clinical Neurosciences, Lausanne University Hospital and University of Lausanne, Lausanne, Switzerland; 5 Department of Neurology, Max-Planck-Institute for Human Cognitive and Brain Sciences, Leipzig, Germany

**Keywords:** Temporal lobe epilepsy, Quantitative magnetic resonance imaging, qMRI, Microstructural tissue property, Voxel-based quantification, Brain plasticity, Mesial temporal lobe

## Abstract

Temporal lobe epilepsy (TLE) is associated with brain pathology extending beyond temporal lobe structures. We sought to look for informative patterns of brain tissue properties in TLE that go beyond the established morphometry differences. We hypothesised that volume differences, particularly in hippocampus, will be paralleled by changes in brain microstructure. The cross-sectional study included TLE patients (*n* = 25) from a primary care center and sex-/age-matched healthy controls (*n* = 55). We acquired quantitative relaxometry-based magnetic resonance imaging (MRI) data yielding whole-brain maps of grey matter volume, magnetization transfer (MT) saturation, and effective transverse relaxation rate R2* indicative for brain tissue myelin and iron content. For statistical analysis, we used the computational anatomy framework of voxel-based morphometry and voxel-based quantification. There was a positive correlation between seizure activity and MT saturation measures in the ipsilateral hippocampus, paralleled by volume differences bilaterally. Disease duration correlated positively with iron content in the mesial temporal lobe, while seizure freedom was associated with a decrease of iron in the very same region. Our findings demonstrate the link between TLE clinical phenotype and brain anatomy beyond morphometry differences to show the impact of disease burden on specific tissue properties. We provide direct evidence for the differential effect of clinical phenotype characteristics on processes involving tissue myelin and iron in mesial temporal lobe structures. This study offers a proof-of-concept for the investigation of novel imaging biomarkers in focal epilepsy.

**Supplementary Information:**

The online version contains supplementary material available at 10.1007/s00429-021-02428-z.

## Introduction

There is mounting evidence for brain pathology extending beyond the temporal lobe in patients with temporal lobe epilepsy (TLE)—one of the most common forms of focal epilepsy. Theoretical work and animal models suggest that TLE-related brain remodeling follows a specific temporal trajectory, with both focal and distributed cortico-subcortical changes that are further modulated in the course of disease (Bernhardt et al. 2013a; Sutula 2004). Most recent reports support the notion of dynamic bidirectional brain anatomy changes related to disease progression, where the initial seizure-induced boost in neurogenesis is followed by gliosis due to depletion of hippocampal stem cells and shift towards astrocytes production (Sierra et al. 2015a, b). Cross-sectional (Bonilha et al. 2004) and longitudinal computational anatomy studies (Bernhardt et al. 2013b) in TLE patients provide indirect evidence for this process, showing hippocampal volume loss in the chronic stages of disease. Given the gap of knowledge about the neurobiology underlying TLE-associated gray matter volume and cortical thickness changes, new magnetic resonance imaging (MRI) techniques provide a window of opportunity to assess pathology related to brain’s iron and myelin homeostasis.

Animal models confirmed the notion that seizures induce axonal and myelin loss in the hippocampus paralleled by dysfunctional axonal sprouting and re-myelination (Savaskan and Nitsch 2001). Under the supposition of seizure-induced neurogenesis rate increase, evidence from animal models shows that newly generated neuronal granule cells migrate into the hilus as far as the hippocampal CA3, where due to abnormal integration into hippocampal networks they start contributing to recurrent excitatory circuits (Scharfman et al. 2000). This seizure-related aberrant network development is followed by hippocampal myelin loss and fiber degeneration in TLE, especially for small diameter axons, demonstrated by animal models and post mortem investigations (Ozdogmus et al. 2009). Correspondingly, human studies demonstrate increased oligodendroglia density and subsequent gliosis in white matter areas adjacent to the seizure onset zone (Kasper and Paulus 2004; Stefanits et al. 2012). Supported by findings showing epilepsy-associated changes of hilar ectopic granule cells (Scharfman et al. 2000) paralleled by changes in myelinated fibers (Luo et al. 2015; Ye et al. 2013), myelin-sensitive neuroimaging techniques would allow to probe microstructural tissue differences in TLE patients.

In the context of TLE, there is strong evidence from animal models about the epileptogenic role of abnormal iron homeostasis in combination with blood–brain-barrier leakage, local inflammation and cellular oxidative stress in the hippocampus (Duffy et al. 2012; van Vliet et al. 2007). Recent studies demonstrate an association between seizure activity, histological and neuroimaging signatures corresponding to pathological iron deposits (Aggarwal et al. 2018). Ferroptosis—the regulated cell death dependent on iron, occurs in the hippocampus following pharmacological-induced TLE in rodents (Ye et al. 2019). Confirmatory for this notion, there is compelling evidence that inhibitors targeting iron homeostasis can prevent hippocampal ferroptosis and ameliorate cognitive impairment associated to TLE (Ye et al. 2019). Human studies find a similar relationship between epilepsy and seizure-dependent inflammation in association to altered iron transfer and iron saturation rates (Tombini et al. 2013; Zhang et al. 2014).

The majority of computational anatomy studies in epilepsy rely on T1- and T2-weighted brain imaging data that are governed by unknown MR contrast contributions, which are a function of the underlying tissue properties. Morphometric features—cortical thickness, surface area or grey matter volume, extracted from this type of MRI data, are heavily dependent on local MR contrast properties that remain unaccounted for across all surface- and voxel-based methods at hand. The missing link between brain tissue properties and resulting morphometry results hinder the straightforward neurobiological interpretation of “spurious” findings (Lorio et al. 2016b). Recent advances in qMRI provide the opportunity to assess in vivo quantitatively specific tissue properties in the healthy and diseased brain with particular focus on myelin, iron and tissue free water content (Draganski et al. 2011; Weiskopf et al. 2013).

Up to date, detection of tissue microstructure pathology in TLE was restricted to histology studies of post-surgery ex-vivo tissue samples that showed altered intracortical myelination and fiber arrangement, particularly in superficial cortical layers (Thom et al. 2000). The combination of state-of-the-art histology and ex-vivo MRI-based morphometry confirmed the spatial correspondence between axonal degeneration of temporopolar white matter and blurring of grey-white matter boundaries (Garbelli et al. 2012). Recent qMRI study in TLE patients demonstrated an ipsilateral cortical and hippocampal increase of the longitudinal relaxation time—a measure sensitive to intracortical myelin, however, with unaccounted contribution of the effects of iron (Bernhardt et al. 2018). This was interpreted as a sign of disrupted fiber architecture that finds correlates in histology specimens of TLE patients.

Our in-vivo study investigated the differences in brain tissue properties associated with TLE clinical phenotype characteristics—disease duration and frequency, beyond the established brain morphometry assessment in TLE patients. To this aim, we acquired qMRI data according to our established relaxometry-based protocol followed by state-of-the-art whole-brain statistical analysis using voxel-based quantification (VBQ) in SPM12s computational anatomy framework (Draganski et al. 2011). We hypothesized that individuals’ seizure frequency and overall duration will correlate with tissue property patterns in hippocampus and associated nodes of limbic circuits.

## Methods

### Participants

For this cross-sectional study, we recruited patients with left-lateralized pharmaco-responsive and -resistant TLE (*n* = 25; 13 females, mean age ± standard deviation 40.9 ± 14.9 years, age range—18 to 69 years old) and sex-/age-matched healthy volunteers (*n* = 55; 28 females, mean age ± standard deviation 36.8 ± 14.6 years, age range 19–72 years old; see Table 1). Handedness was estimated based on the Edinburgh Handedness Inventory and classified into right-handedness dependent on scores superior to + 40, ambidexter—with scores between − 40 and + 40 and left handedness—scores inferior to − 40 (Oldfield 1971).

**Table 1:** Demographic and clinical information of study participants

	TLE	C
#	25	55
Sex		
F	13	28
M	12	27
Age [years]	41 [15]	37 [15]
Handedness [#]		
Right	19	44
Left	1	2
Ambi-dexter	5	9
Age at epilepsy Onset [years]	28.2 [16.6]	
Disease duration [years]	12.7 [11.4]	
Seizure generalized [#] [life]	15.8 [7.9]	
Seizure free [months]	45.9 [22.0]	
Seizure freq focal [years]	11.4 [28.8]	
AED tried [#]	1.6 [0.9]	
TIV [l]	1.56 [0.18]	1.58 [0.17]

Displayed values are means with standard deviation, given in squared brackets [] for left-lateralized TLE and healthy controls

*TLE* temporal lobe epilepsy, *C* healthy controls, *#* number of, *AED* antiepileptic drug treatment, *TIV* total intracranial volume, *f* female, *m* male, *l* liter

The decision to restrict the analysis to only left lateralized TLE aimed to differentiate unilateral versus bihemispheric effects. The protocol was approved by the local Ethics Committee and prior to study inclusion we obtained written informed consent from each participant. All procedures were performed in accordance with national and international guidelines.

The diagnosis of mesial TLE followed the criteria of the International League Against Epilepsy (Berg et al. 2010) including (i) clinical aspects of seizures such as semiology, onset and history; (ii) standard and/or long-term video-electroencephalography (EEG) and (iii) neuro-radiological assessment. Clinical 3 Tesla MRI was obtained according to established protocols (Wellmer et al. 2013) at University Hospital CHUV Lausanne, and reviewed by a neuroradiologist with special expertise in epileptology (for pharmaco-responsiveness and -resistance, see “Results” section). The evaluation of the lateralization of the epileptogenic seizure onset zone depended on seizure semiology, evidence of unilateral epileptiform activity on EEG, and MRI findings. Patients without strong evidence for lateralization of the seizure onset zone or with a bilateral, lateral temporal or extra-temporal epileptogenic focus (focal cortical dysplasia or lateral temporal sclerosis) were excluded from subsequent analysis, as we aimed at investigating structural changes in temporal lobe epilepsy including a clear epileptogenic focus. Additional exclusion criteria included a history of psychogenic non-epileptic seizures, autoimmune etiologies of epilepsy, history of traumatic brain injury, evidence of ischemic or hemorrhagic brain lesions, tumors, or neuro-radiological diagnosis of brain pathology beyond hippocampal and mesial temporal lobe sclerosis.

Following radiological evaluation criteria, our cohort consists of 5 patients with lesional epilepsy (3—with hippocampal sclerosis, 1—with hippocampal dysplasia, and 1—with hippocampal malrotation), while for the remaining 20 patients, clinical MRI did not show any evident lesion. The definition of epileptogenic focus lateralization was based on inter-ictal epileptiform discharges. 21 patients were drug responsive, i.e. seizure-free since more than 5 years, 4 patients were resistant to antiepileptic drug treatment, dependent on consensus definition of the International League Against Epilepsy (Kwan et al. 2010).

To quantify epilepsy severity, we documented and calculated the number of auto-reported generalized tonic–clonic seizures across life time and of focal seizures without generalization within the last 12 months. The date of MRI acquisition is used as endpoint for the time interval of interest. We refrained from including reports of focal seizures without generalization dating back more than 12 months ago given the expected lack of precision, particularly for non-generalized seizures. Only 3 out of the 25 patients presented focal seizures without generalization. 1 of the 3 patients did not have any focal seizure within 12 months before data acquisition.

Disease duration was calculated as the time span between the first seizure and MRI data acquisition. The seizure-free interval represented the time between the last seizure and MRI data acquisition. 6 patients were in remission with a free interval of more than 5 years for generalized seizures and 4 out of these 6 patients were in remission for focal seizures too.

### Magnetic resonance imaging data acquisition

We acquired quantitative MRI (qMRI) data on a 3 Tesla MRI system (Magnetom Prisma, Siemens Medical Systems, Germany) using a 64-channel radio-frequency receive head coil and body coil for transmission. The protocol consisted of three multi-echo 3D fast low angle shot (FLASH) with proton density (TR/α = 24.5 ms/6°), T1 (TR/α = 24.5 ms/21°), and magnetization transfer (MT) (TR/α = 24.5 ms/6°)-weighted contrasts (Helms et al. 2008) using a field-of-view of 240 × 256 × 176 mm along with the A-P, H-F and L-R directions and isotropic voxel sizes of 1 × 1 × 1 mm (Weiskopf et al. 2013). We used parallel imaging along the phase encoding (acceleration factor 2, GRAPPA image reconstruction) and Partial Fourier 6/8 in the partition direction to speed up data acquisition. Data was acquired for mapping inhomogeneities of the radio-frequency transmit field B1 using a 3D Echo Planar Imaging spin-echo/stimulated-echo method (Lutti et al. 2012). The acquisition parameters were as follows: TR = 500 ms, TE = 39.06 ms, TM = 31.2 ms, FOV = 192 × 256 × 192 mm along with the A-P, H-F and L-R directions, image resolution = 4mm^3^. 2D dual-echo field map data was acquired to correct for image distortions in the EPI data using the SPM field map toolbox (Hutton et al. 2012). The total acquisition time was 27 min.

### qMRI map calculation and image processing

For data preprocessing and analysis we use the established computational anatomy framework of SPM12s (www.fil.ion.ucl.ac.uk/spm/software/spm12) with voxel-based morphometry (VBM) and VBQ (Draganski et al. 2011) running under Matlab 7.13 (Mathworks Inc., Sherborn, MA, USA). Regression of the log-signal from the 8 proton density-weighted echoes was used to calculate the R2* maps. Signals of six equidistant bipolar gradient echoes were averaged to augment the signal-to-noise ratio (Helms and Dechent 2009) before calculating the MT saturation maps (Weiskopf et al. 2013). All qMRI data were corrected for radiofrequency transmit inhomogeneities using the B1 maps (Lutti et al. 2014).

### Image processing

For automated brain tissue classification and subsequent regional volume calculation, we used the MT saturation maps with SPM12s default settings of the “unified segmentation” framework (Ashburner and Friston 2005) and enhanced tissue probability maps providing higher sensitivity for detection of subcortical structures (Lorio et al. 2016a). We estimated spatial registration parameters to standard Montreal Neurological Institute space for volume and qMRI maps with SPM12s diffeomorphic algorithm based on exponentiated lie algebra (DARTEL) (Ashburner 2007). The GM probability maps for voxel-based morphometry analysis were scaled with the corresponding Jacobian determinants, whilst MT and R2* maps underwent the previously described weighted averaging (Draganski et al. 2011) to preserve the total signal average. Prior to statistical analysis all maps were spatially smoothed using an isotropic 3D Gaussian convolution kernel of 6 mm full-width-at-half-maximum.

### Statistical analysis

**Fig. 1:**
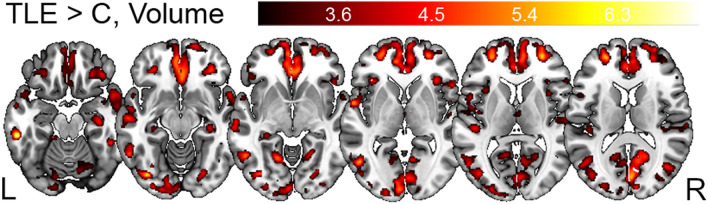
Grey matter volume differences between left TLE patients and healthy controls. Statistical parametric maps (SPM) of grey matter volume differences between patients with left-lateralized TLE and healthy controls. SPMs displayed on axial T1-weighted image in standard MNI space. For visualization purposes SPMs thresholded at *p* < 0.001, uncorrected for multiple comparisons. *TLE* temporal lobe epilepsy, *C* healthy controls, *L* left, *R* right hemisphere

**Fig. 2:**
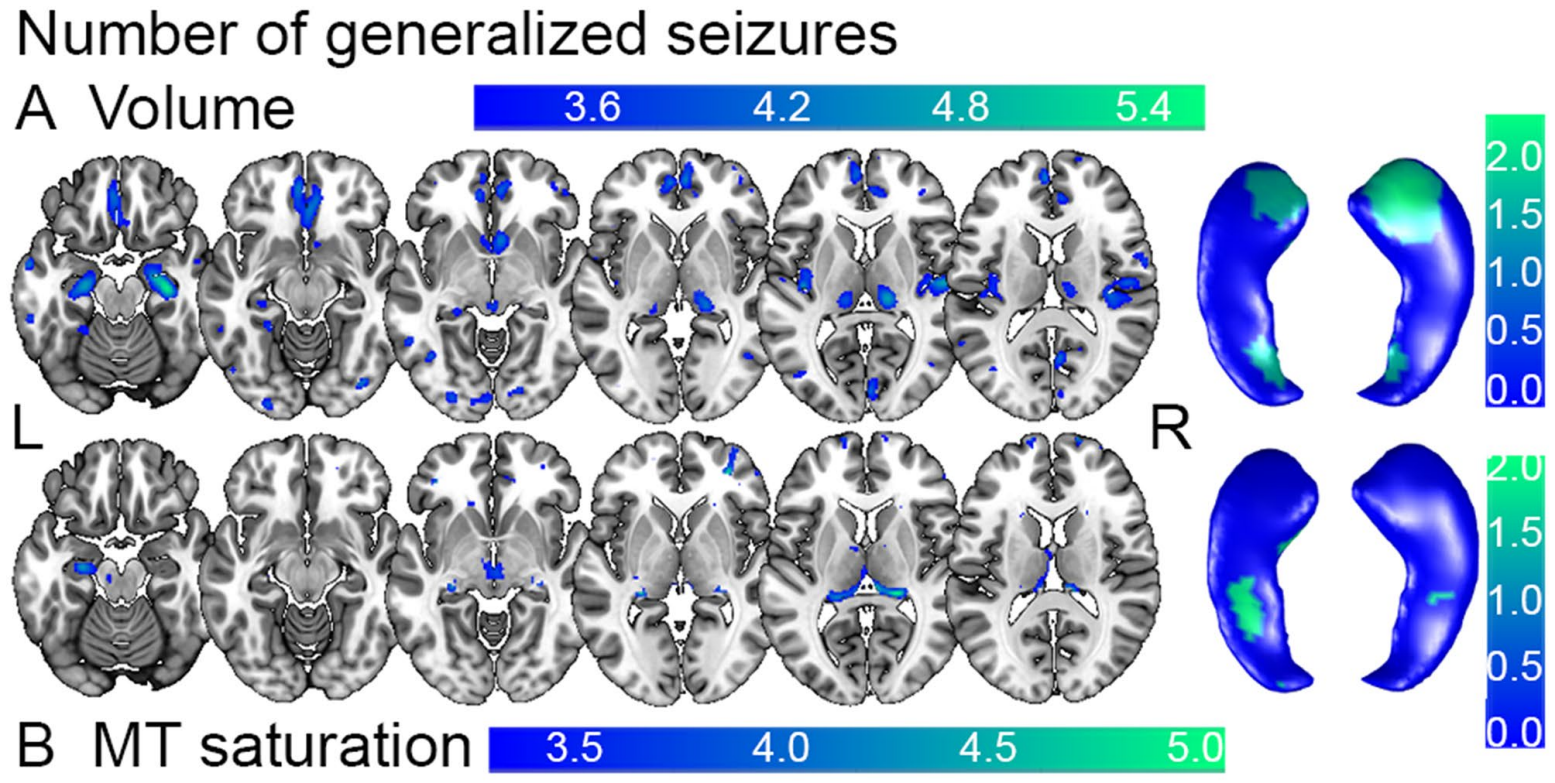
Correlation between seizure frequency and brain anatomy in left TLE patients. Statistical parametric maps (SPM) of positive correlation between **A** volume estimates and number of generalized seizures and between **B** myelin estimates based on MT saturation intensity values and number of generalized seizures in left TLE. Whole-brain results of SPM *T* statistics projected on axial T1-weighted image in standard MNI space on left side and on bilateral hippocampal surfaces on right side. For visualization purposes SPMs thresholded at *p* < 0.001, uncorrected for multiple comparisons. *TLE* temporal lobe epilepsy, *MT* magnetization transfer (saturation), *L* left, *R* right hemisphere

We concatenated whole-brain volume, MT saturation and R2* maps of left-lateralized TLE patients and healthy participants within a single multi-parametric ANOVA design to test for between-group differences (Fig. 1, Table 2A). Age, sex and total intracranial volume (TIV) were included as additional variables to control for their specific effects on brain anatomy. Using whole-brain and region-of-interest analyses, we tested correlations between brain morphometry, tissue properties and clinical phenotype—number of generalized seizures, disease duration and seizure-free interval preceding the MRI data acquisition (Figs. 2 and 4, Table 2B). For the post-hoc region-of-interest analysis testing the correlation between hippocampus volume/myelin content, seizure frequency and disease duration, we calculate the optimal weighted averages of TLE individuals’ hippocampal volumes using the first eigenvector as provided by SPM12’s volume-of-interest function. The spatial extent of the hippocampus was defined using the neuromorphometrics atlas (probabilistic and maximum probability tissue labels—derived from the “MICCAI 2012 Grand Challenge and Workshop on Multi-Atlas Labeling”, www.neuromorphometrics.com/2012_MICCAI_Challenge_Data.html).

**Table 2:** Main effects of disease (A) and Correlation between brain structure, microstructural tissue properties and clinical phenotype in left TLE (B)

MR modality	Clinical phenotype	Correlation	Brain region	Hemi-sphere	MNI coordinates [mm]			Cluster size	*p*_FWE-corr_	*Z* score
			Cluster max	L/R	*x*	*y*	*z*	[# voxel]		
A										
Volume	L TLE > C		Middle temporal gyrus	L	− 60	− 18	− 9	681	0.004	4.76
			Cerebellum exterior, posterior lobe	R	26	− 83	− 29	2351	0.000	4.71
			Frontal operculum	R	50	18	− 2	724	0.003	4.62
			Parietal operculum	R	51	− 30	21	1016	0.000	4.54
			Hippocampus	R	33	− 21	− 17	399	0.048	4.47
			Postcentral gyrus	R	60	− 11	38	484	0.022	4.42
			Frontal operculum	L	− 33	26	3	523	0.016	4.31
			Hippocampus	L	− 29	− 36	− 5	188	0.031	3.96
B										
Volume	#Seizure	+	Hippocampus	R	30	− 17	− 20	1361	0.000	5.52
			Cerebellum Exterior, posterior lobe	R	12	− 50	− 57	4168	0.000	5.39
			Cerebellum Exterior, posterior lobe	L	− 26	− 75	− 48	2956	0.000	5.16
			Transverse temporal gyrus	R	51	− 15	9	1475	0.000	4.94
			Hippocampus	L	− 24	− 17	− 20	985	0.000	4.65
			Thalamus proper	R	15	− 26	6	666	0.005	4.64
			Posterior Insula	L	− 39	− 18	9	668	0.005	4.60
			Orbitofrontal cortex, anterior cingulate cortex	L	− 5	44	− 15	3349	0.000	4.56
MT	#Seizure	+	Thalamus proper	R	17	− 32	− 9	1309	0.000	4.73
Saturation			Middle frontal gyrus	R	34	18	27	205	0.040	4.17
			Hippocampus	L	− 26	− 17	− 17	219	0.035	4.08
R2*	Disease duration	+	Inferior temporal gyrus	L	− 65	− 30	− 23	753	0.003	6.37
			Hippocampus	L	− 20	− 34	− 6	14,823	0.000	5.91
			Anterior insula	R	44	0	− 2	2319	0.000	5.51
			Putamen	R	19	14	− 12	2862	0.000	5.40
			Cerebellum, posterior lobe	L	− 23	− 44	− 42	471	0.029	4.79
	Seizure-free interval	–	Fusiform gyrus	R	42	− 36	− 30	481	0.027	5.09

Comparison between grey matter volume in left TLE cohort and C. Parameters refer to local maxima within each cluster at the statistical threshold of *p* < 0.05, cluster level, family-wise error corrected for multiple comparisons [see Fig. 1]. Parameters refer to local maxima within each cluster at the statistical threshold of *p* < 0.05, cluster level, family-wise error corrected for multiple comparisons [see Figs. 2, 4]

*TLE* temporal lobe epilepsy, *C* healthy control volunteers, *L* left, *R* right, *#* number of, *MT* magnetization transfer saturation, R2* effective transverse relaxation rate, *AED* antiepileptic drug treatment, *[−]* negative and *[* +*]* positive correlation

**Fig. 3:**
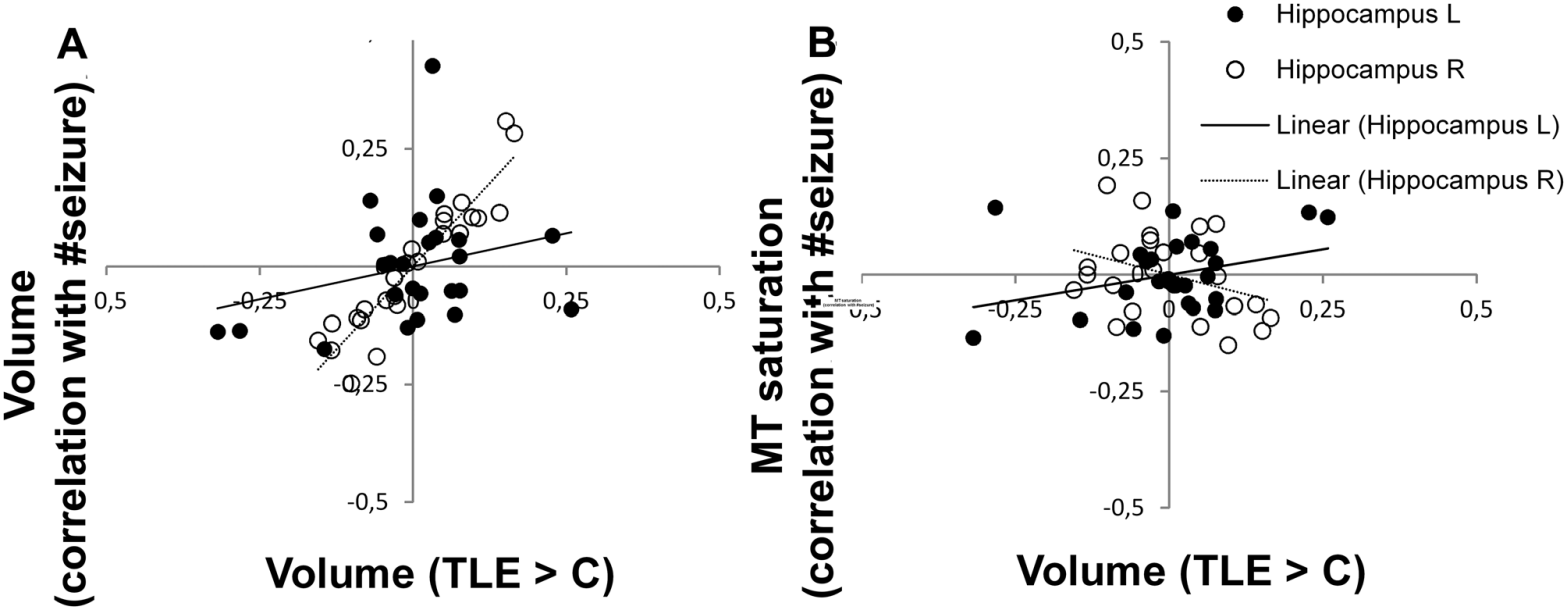
Correlation between hippocampal grey matter volume and number of generalized seizures. Hippocampal volume estimates in left TLE patients (*x* axis) plotted against **A** number of generalized seizures (*y* axis); **B** MT saturation (*x* axis) and number of generalized seizures (*y* axis). Significant partial correlation for ipsilateral left hippocampus (*p* = 0.023) and for contralateral right hippocampus (*p* < 0.0001), correlation of MT parameters not significant (*p* > 0.05). Optimal weighted averages of hippocampal volumes extracted from left and right hippocampus. *TLE* temporal lobe epilepsy, *C* healthy controls, *L* left, *R* right, *#* number of

We report significant results at a statistical threshold of *p* < 0.05 after family-wise error (FWE) correction for multiple comparisons (Table 2) and as trends when below *p* < 0.001, uncorrected for multiple comparisons (Figs. 1, 2 and 3).

## Results

### Demographic and clinical phenotype

There were no significant sex-, age- or TIV-related differences between healthy controls and TLE patients. Five patients were ambidexter and one had a left-hand dominance (Table 1). The remaining 19 TLE patients were right-handed. The handedness (*p* = 0.54) and sex distribution (*p* = 0.34) of TLE patients were comparable to healthy controls.

### Main effects of disease

We report a larger hippocampal volume bilaterally in TLE patients, additionally to operculum bilaterally, ipsilateral middle temporal gyrus and contralateral cerebellar and post-central regions (Fig. 1, Table 2A). There were no significant MT and R2* differences in the group comparison.

### Seizure activity

The whole-brain VBM analysis showed a positive correlation between seizure frequency, measured by number of generalized seizures, and the volume of hippocampus bilaterally (Fig. 2A, Table 2B). We denote the higher statistical significance confined to the anterior hippocampus. We point out that the region denoted as hippocampal tail in the voxel-based analysis may include also parts of the fornix. There was a similar effect for the contralateral transverse temporal gyrus and thalamus, the ipsilateral posterior insula, orbito-frontal cortex, anterior cingulate cortex and cerebellar posterior lobes bilaterally. The VBQ analysis demonstrated spatially overlapping results with a positive correlation between MT saturation and seizure activity in the ipsilateral hippocampal body, bilateral thalamus and the contralateral transverse temporal gyrus (Fig. 2B, Table 2B).

The post-hoc region-of-interest analysis centered on the hippocampus (Fig. 1) confirmed the correlation between number of generalized seizures (Fig. 2) with hippocampus volume bilaterally with emphasis on the right hippocampus (Figs. 1 and 3A). The MT saturation analysis did not show significant correlations (Fig. 3B).

### Disease progression

**Fig. 4:**
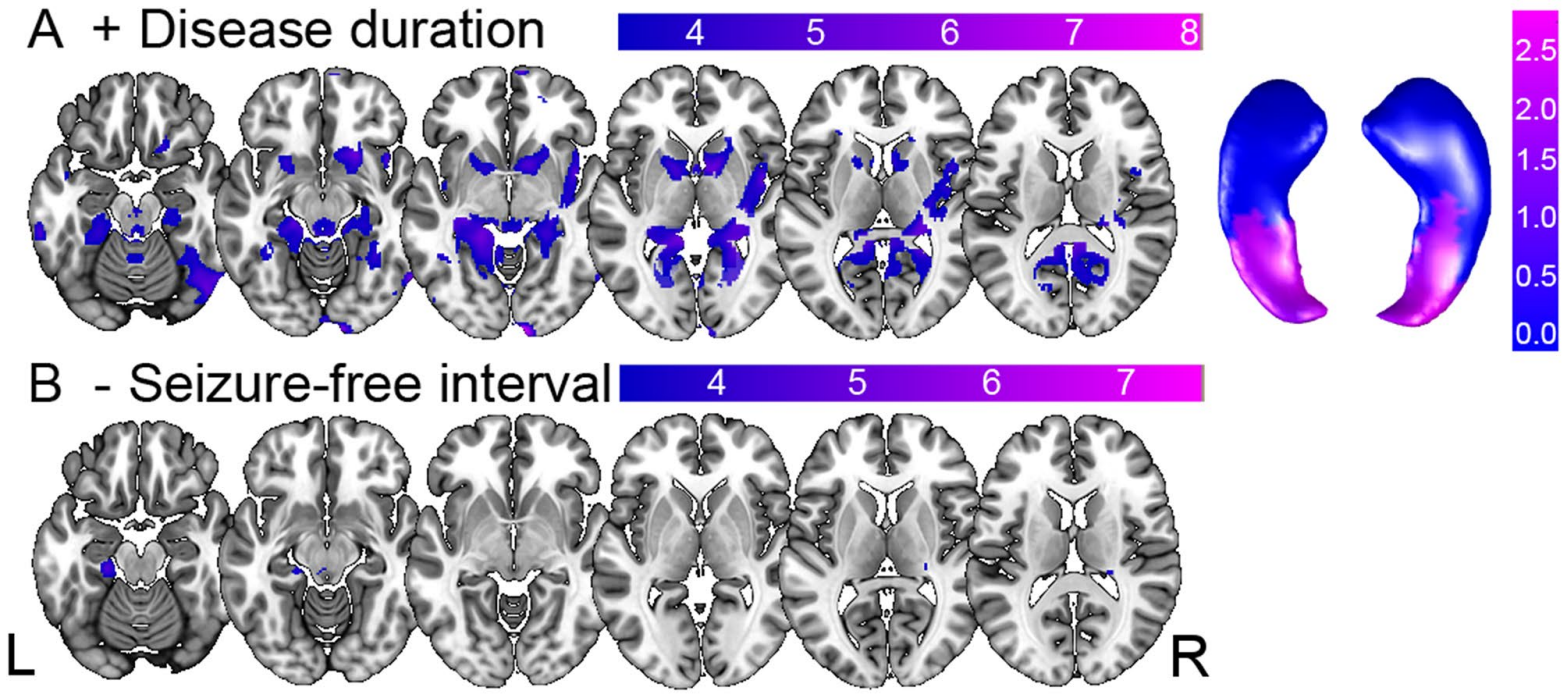
Correlation between disease progression, seizure-free interval and iron content. Statistical parametric maps of positive and negative correlation between effective transverse relaxation rate R2* intensity values and **A** disease duration in years (with values projected on bilateral hippocampal surfaces left) and **B** the seizure-free interval in months in left TLE. Results displayed on T1-weighted image in MNI space, statistical threshold at *p* < 0.001, uncorrected for multiple comparisons. *[−]* negative, *[* +*]* positive correlation, *L* left, *R* right

The VBQ analysis revealed a positive correlation between disease duration and R2* increase, indicative for iron content of the ipsilateral hippocampus, the transverse temporal gyrus and cerebellum as well as contralateral insula and basal ganglia (Fig. 4A). In contrast, seizure-free intervals correlated negatively with R2* values in the ipsilateral parahippocampal gyrus and the contralateral fusiform gyrus (Fig. 4C).

## Discussion

Our study demonstrates a specific pattern of volume and brain tissue property differences in TLE patients that were associated with individuals’ disease duration and seizure frequency. The observed hippocampal volume expansion in TLE patients is paralleled by MT saturation increase in the very same region. Differences in mesial temporal lobe volume and tissue properties were associated with individuals’ seizure activity. Our qMRI multi-parameter approach extends previous morphometry findings in patients with TLE to show concomitant microstructure differences that are interpreted in the context of underlying neurobiological processes.

### Seizure activity associated with differences in regional volume and myelin content

Our main finding is that seizure activity and TLE disease duration impact hippocampal microstructure beyond the already known effects on volume and shape. The effects are bilateral, thus independent from seizure focus localization. This finding is at odds with the imaging neuroscience literature that reports volume loss interpreted in the context of hippocampal sclerosis, which can be seen in chronic cases even with the naked eye. There are two lines of argumentation that support the notion of initial volume increase in the early phase of TLE—one related to the underlying neurobiological processes and another—to methodological and interpretational issues of computational anatomy studies using T1-weighted imaging.

We interpret the hippocampal volume increase in the early phase of TLE as indicative for seizure-induced boost of neurogenesis potentially paralleled by mossy fiber sprouting and changes in density/persistence of hilar basal dendrites. This assumption is based on the cumulating evidence from animal TLE models (Sierra et al. 2015a, b) and from findings in both animal model (Madsen et al. 2000; Olesen et al. 2017) and investigations in patients treated with electro-convulsive therapy (Dukart et al. 2014). A particular detail – the gradient of seizure impact along the longitudinal hippocampal axis with stronger effects in the anterior hippocampus, seen after electro-convulsive therapy (Dukart et al. 2014) and in our study—in the correlation analysis with seizure frequency, lends further credibility to our findings. For the chronic phase of TLE, hippocampal atrophy, due to depletion of the stem cell pool and ongoing asymmetric cell division with predominant generation of astrocytes (Sierra et al. 2015a, b), can explain the widely accepted radiological and histology findings.

The second line of argumentation addressing the controversy in the computational anatomy literature about TLE-induced brain structure differences in patients (Bernhardt et al. 2013b; Briellmann et al. 2002; Coan et al. 2014; Holtkamp et al. 2004; Mueller et al. 2006) stems from the fact that the majority of studies use T1-weighted imaging for assessment of volume or cortical thickness. Independent from the methods used for feature extraction and statistical analysis—i.e. surface- or voxel-based, the fact that the MR contrast is dependent on underlying tissue properties and that T1-weighted MRI protocols are not informative with respect to these changes, prompted a revision of the established neurobiological interpretation. Recent evidence about the occasional “erroneous” interpretation of nature and directionality of morphometric changes is provided in the context of brain maturation (Natu et al. 2019) and ageing (Lorio et al. 2016b; Taubert et al. 2020). Our volume estimates are based on qMRI data, which renders them more robust to the underlying tissue property characteristics in the context of TLE (Lorio et al. 2016b).

The novelty of our VBQ approach comes from the analysis of parameters indicative for tissue property differences, beyond and above the morphometry findings. The observed positive correlation between MT saturation and seizure frequency restricted to the hippocampus ipsilateral to seizure onset can be interpreted in the context of neurogenesis and related biological phenomena. Given the fact that MT saturation is based on differences in macromolecular content rather than in molecular mobility, it allows for the distinction of densely packed gray matter from normal gray and white matter but is also sensitive to tissue’s myelin content (Draganski et al. 2011; Helms et al. 2008). We interpret the MT saturation findings as correlates of axonal sprouting of existing and newly generated neurons in the ipsilateral to seizure onset hippocampus, most probably secondary to seizures (Savaskan and Nitsch 2001) or as an additional marker of increased neurogenesis,—i.e. cellular density.

### Disease progression related to iron concentration estimates

The observed positive correlation between the transverse relaxation rate—R2* and disease duration is interpreted as iron accumulation, inflammation, edema or blood leakage. Iron accumulation confined to the mesial temporal lobe of TLE patients is supported by previous studies suggesting a seizure-dependent activation of an inflammatory cascade involving IL-6 and TNF-α cytokines leading to altered iron transfer and saturation (Ikeda 2001; Tombini et al. 2013; Zhang et al. 2014). In patients with epilepsy, transferrin saturations are increased as a surrogate marker for iron overload (Ikeda 2001) and the presence of altered iron suggests dysfunctional regeneration after seizure. In line with a previous study, our findings support the interpretation that a seizure-induced increase of the permeability of the blood–brain-barrier leads to extravasation of blood components and hemoglobin with the consequence of ferritin overexpression in the hippocampus (Gorter et al. 2005). Another argument for the role of iron in TLE patients is the fact that in the animal model, iron chelators ameliorate seizure-induced mitochondrial oxidative stress, excitotoxic neuronal injury and hippocampal cell loss (Liang et al. 2008). Alternatively, one can assume that volume and qMRI parameters could be influenced by vasogenic or cytotoxic edema after generalized seizures (Scott et al. 2006). Given the mean of seizure-free intervals of 46 months with a minimum interval of two months, the probability of seizure-induced impact on the presented results is negligible.

We acknowledge potential study limitations that could impact our results and interpretations. The cross-sectional nature of the study limits our inferences to correlation rather than causation. Given the novelty of the neuroimaging approach, we focused on a rather small clinical cohort with unilateral left-sided seizure onset. Epilepsy severity is inferred from subjective reports of seizures with secondary generalization. Subjective seizure diaries only give a restricted insight into individual real seizure activity. The current approach focuses exclusively on seizures with generalization as determinants of temporal lobe changes. Focal seizures without generalization were not considered in the analysis. Histological interpretation of the MT and R2* maps are based on references studies (Helms et al. 2008), however building on validated biophysical models.

## Conclusion

Our combined VBM/VBQ study offers additional neurobiological interpretation linking disease duration, seizure frequency, brain volume and tissue properties in pharmaco-responsive and -resistant TLE patients. We interpret our results as evidence for a seizure-induced boost of neurogenesis and axonal sprouting associated with myelination, which is followed by a continuous, but reversible accumulation of iron in the mesial temporal lobe. Non-invasive assessment of brain tissue properties could become relevant for the clinical evaluation and outcome prediction in TLE.

## Key Points

- qMRI biomarkers of brain tissue microstructure are correlates of TLE clinical phenotype providing insight into microscopic processes underlying disease
- We investigate qMRI in TLE patients and healthy controls by MT saturation and effective transverse relaxation rate, biomarkers of myelin and iron concentration
- Seizure activity correlates with MT saturation measures in ipsilateral hippocampus paralleled by volume differences in same area
- Disease duration correlates positively with R2* measures in mesial temporal lobe, while seizure freedom is associated with decrease of R2* in very same region
- Clinical phenotype characteristics are linked to processes involving myelination and iron differences in mesial temporal lobe structures

## Supplementary Information

Below is the link to the electronic supplementary material.

Supplementary file1 (PNG 41 KB)

## Data Availability

The data is currently not available online. Anonymized data is available on demand.

## Abbreviations

EEG: Electroencephalography
FEW: Family-wise error
GM: Grey matter
MT: Magnetization transfer
SPM: Statistical parametric mapping
TLE: Temporal lobe epilepsy
qMRI: Quantitative magnetic resonance imaging
VBM: Voxel-based morphometry
VBQ: Voxel-based quantification

## Code availability

The used code is publicly available including: SPM 12 (www.fil.ion.ucl.ac.uk/spm/software/spm12) with voxel-based morphometry (VBM) and the current version of the VBQ toolbox (https://hmri-group.github.io/hMRI-toolbox/) running under Matlab 7.13 (Mathworks Inc., Sherborn, MA, USA).
