# Supplementary figures and images for: Clinical phenotype modulates brain’s myelin and iron content in temporal lobe epilepsy
Source: Brain Struct Funct. 2021 Nov 24;227(3):901–11. doi: 10.1007/s00429-021-02428-z (PMC8930791; doi:10.1007/s00429-021-02428-z)

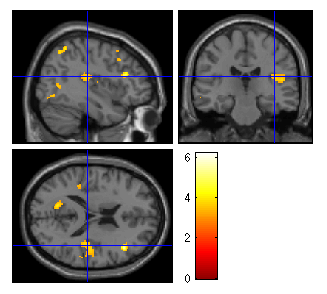

Supplement: Supplementary file 1 — Supplementary file1 (PNG 41 KB) [file 429_2021_2428_MOESM1_ESM.png]
